# Increased Urinary Cadmium Levels in Foreign-Born Asian Women—An NHANES Study of 9639 U.S. Participants

**DOI:** 10.3390/ijerph19042170

**Published:** 2022-02-15

**Authors:** Anthony Milki, Deanna Wong, Chloe Chan, Sarita Sooklal, Daniel S. Kapp, Amandeep K. Mann

**Affiliations:** 1 School of Medicine and Health Sciences, The George Washington University, Washington, DC 20037, USA; anthonymilki@gwmail.gwu.edu; 2 Department of Obstetrics and Gynecology, UCLA Medical Center, Los Angeles, CA 90095, USA; dhwong@mednet.ucla.edu; 3 California Pacific Medical Center, San Francisco, CA 94109, USA; chloemchan1@gmail.com; 4 Keck School of Medicine of USC, University of Southern California, Los Angeles, CA 90033, USA; sooklal@usc.edu; 5 Department of Radiation Oncology, Stanford University School of Medicine, Stanford, CA 94305, USA; dskapp@stanford.edu; 6 Palo Alto Medical Foundation Research Institute, Palo Alto, CA 94301, USA

**Keywords:** environmental toxins, cadmium, social determinants of health, racial disparities, occupational health, environmental exposure, women’s health

## Abstract

The purpose of this study was to determine the disparities and trends in demographics, social behaviors, and occupations for cadmium exposure in the U.S. Data were obtained from the NHANES database from 2007 to 2016. Analysis of variance tests were used to compare the association of the geometric mean values of urinary cadmium levels and various demographic and behavioral characteristics. We also conducted multivariable logistic regression while adjusting for these factors to determine the risk of toxic urinary cadmium levels (≥2 µg/g) across various patient characteristics. Of the 9639 participants, 52.8% were ≥45 years old, 51.7% female, and 48.3% male. White, Black, Mexican American, other Hispanic, and Asian comprised 66.4%, 11.5%, 8.7%, 5.8%, and 5.5%, respectively. Over 82% of participants were U.S. born. A total of 19.6% were current smokers. On multivariate analysis, older age (OR: 8.87), current smoking (OR = 5.74), Asian race (OR = 4.52), female sex (OR = 4.32), and foreign nativity (OR = 1.83) were significantly associated with higher cadmium levels. Older, Asian, foreign-born females showed a measurement of 0.69 μg/g, a value more than three-fold the sample population’s mean of 0.20 μg/g. A trend analysis demonstrated a cadmium level decrease over time (OR = 0.47). Asians had the highest urinary cadmium levels, especially older, foreign-born females. Smoking and poverty were also associated with significant elevations in cadmium levels.

## 1. Introduction

Cadmium is one of many heavy metals that can accumulate in the kidneys and liver throughout an individual’s life. Dietary, environmental, and occupational sources of cadmium are the main contributors to daily cadmium exposure levels [1], and the Occupational Safety and Health Administration (OSHA) has set the permissible exposure limit to 5 µg/m^3^ (fumes) in workplaces to protect the health of people that are occupationally exposed to the metal. Additionally, the National Institute of Occupational Safety and Health (NIOSH) set an immediate Dangerous to Life and Health level (IDLH) of cadmium, which is 9 mg/m^3^ [2]. Chronic, low-level cadmium exposure can lead to various detrimental outcomes including depression, cardiovascular disease, reduced renal function, early menopause and premature ovarian insufficiency, osteoporosis, and cancers [3,4,5,6]. Considering the severity of the diseases, it is important to understand the factors associated with excessive cadmium exposure.

Prior studies on blood cadmium levels showed that older age, females, current smokers, and blue-collar occupations are factors associated with increased levels [7,8,9]. However, blood levels are a less accurate representation of long-term cadmium accumulation compared to urinary measurements [10]. Several studies on urinary cadmium levels only evaluated reproductive-age women and did not investigate detailed factors and trends in cadmium exposure over time [8].

In this current report, we studied 9639 participants from an United States national survey database to determine demographic factors and social behaviors associated with increased urinary cadmium levels. These factors included age, nativity, race, sex, income level, body mass index, smoking status, and longest-held occupation. Furthermore, an intersectionality analysis was conducted to identify subgroups at the highest risk for cadmium exposure and a trends analysis was performed to study exposure over time.

## 2. Materials and Methods

### 2.1. Data Source and Study Sample

**Figure 1:**
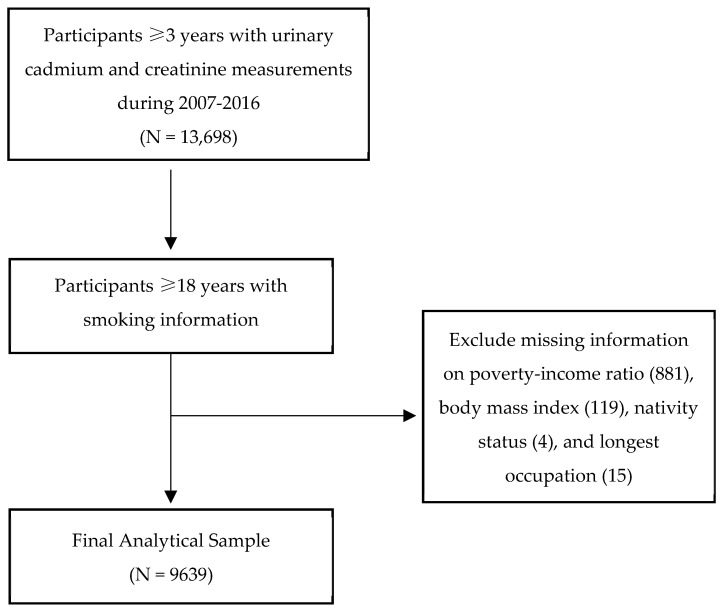
Participant selection.

Our sample data were obtained from the National Health and Nutrition Examination Survey (NHANES) from 2007 to 2016 and included 9639 participants. NHANES is a database that was created by the Centers for Disease Control and Prevention (CDC) in order to examine the participants’ health and nutrition information. It collects data in 2-year cycles (beginning in 1999) through home interviews and mobile examination centers [11]. Since NHANES oversamples participants who are 60 years or older, Black, Hispanic, and Asians (since 2011 for the latter), we incorporated multistage, stratified, clustered probability, and sampling weights of the U.S. population. Between 2007 and 2016, a total of 13,698 participants three years or older had urinary cadmium and creatinine measurements. Because smoking information was only collected from those participants 18 years or older, our data were further restricted to this age group (N = 10,658). We excluded missing data information on poverty–income ratio (881), body mass index (119), nativity status (4), and longest occupation (15), which resulted in a sample size of 9639 participants (see Figure 1).

### 2.2. Covariates

Participants’ age group was dichotomized using the median age of 45 years. Nativity status was based on the participants responding to ‘In what country were you born?’. Those who responded not being born in the U.S. were categorized as ‘foreign.’ Race was categorized as White, Black, Mexican American, other Hispanic, Asian, or other. Gender was grouped as ‘male’ and ‘female.’ Participants’ body mass index (BMI) was collected through a mobile examination center. According to the CDC, BMI was categorized as under-weight (<18.5 kg/m^2^), normal (18.5–24.9 kg/m^2^), overweight (25.0–29.9 kg/m^2^), and obese (≥30.0 kg/m^2^) [12]. To determine income levels for our study, poverty–income ratio (PIR) was used, which is the ratio of family income to poverty threshold to incorporate economic inflation adjusted by year. Using the U.S. Census of income categories, income levels were defined as poverty: PIR <1; low income: 1.0 ≤ PIR < 2.0; middle income: 2.0 ≤ PIR < 4.0; and high income: PIR ≥4.0. Smoking status was categorized as current, former, and non-smokers. Longest-held occupation in our study includes the job that was held the longest based on a standardized NHANES database question (“Thinking of all the paid jobs or businesses you ever had, what kind of work were you doing the longest? e.g., electrical engineer, stock clerk”), with similar occupations categorized together as specified in Table 1 [11,13].

### 2.3. Outcome Measurements

Urinary cadmium level measurements were obtained from a single urine specimen by the CDC laboratory using an inductively coupled plasma-mass spectrometry (ICP-MS), which is instrumental in detecting metals. Results of urinary cadmium were reported in nano-grams per milliliter. Similarly, urinary cadmium was obtained using the methodology described above using the same urine specimen and reported results in milligrams per deciliter. The ICP-MS method can sensitively detect the exposure risk of toxic elements found in the participants’ urine specimens. For results below the detection limit, NHANES corrected the value by dividing the detection limit of urinary cadmium by the square root of 2. All urine specimens were processed, stored, and shipped to the National Center for Environmental Health, CDC for analysis [14]. To account for the effect of urinary dilution, urinary cadmium was adjusted using the concentration of urinary creatinine. This adjustment was made by dividing urinary cadmium (ng/mL) by urinary creatinine (mg/dL) and multiplying by 100 to obtain creatinine-adjusted urinary cadmium (µg/g).

### 2.4. Statistical Analysis

The geometric means of creatinine-adjusted urinary cadmium levels were calculated for each subgroup since the outcome of interest was not normally distributed. We found extreme values of urinary cadmium—7.56, 5.9, and 5.49 ug Cd/g creatinine—that, once removed, did not alter the results. Patients with these elevated cadmium levels were therefore included in the analysis. Analysis of variance (ANOVA) tests were used to compare the log-transformed geometric mean of creatinine-adjusted urinary cadmium levels among the subgroups for each factor. Urinary cadmium level trends were calculated with regard to various demographic factors.

To understand toxic or high risk exposure of urinary cadmium, we dichotomized the dependent variable using the cut-off 2 µg Cd/g creatinine. According to the International Union of Pure and Applied Chemistry (IUPAC) technical report on the risk assessment of cadmium exposure on human health, cadmium levels ≥2 µg Cd/g creatinine can cause harmful side effects to the bone, kidneys, and other organs. Thus, we conducted multi-variable logistic regression to determine the association of high risk urinary cadmium levels (≥2 µg Cd/g creatinine) and the participants’ characteristics, adjusting for time-period, age group, nativity status, race/ethnicity, gender, income level, body mass index, smoking status, and longest occupation held. Furthermore, since detailed information on the longest occupation was collected until 2014, the multivariate only included analysis between 2007 and 2014. A subset multivariate analysis was conducted to examine the impact of cadmium exposure in Asians from 2011–2016. To determine the effects of cadmium on certain sub-populations, we conducted intersectionality analysis of the most high-risk groups based on our geometric mean analysis.

All statistical analyses were conducted using SAS Enterprise Guide 7.1 (SAS Institute Inc., Cary, NC, USA). Since our data came from a public use file and did not contain any identifying information of the participants, this study was exempt from IRB approval.

## 3. Results

### ANOVA and Multivariable Regression Findings

Of the 9639 participants, 52.8% were ≥45 years old, 51.7% were female, and 48.3% were male. White, Black, Mexican American, other Hispanic and Asian comprised 66.4%, 11.5%, 8.7%, 5.8%, and 5.5%, respectively. Over 82.1% participants were U.S. born. A total of 19.6% were current smokers, 23.9% were former smokers, and 56.4% were nonsmokers. BMI categorizations were underweight (2.6%), normal (29.8%), overweight (32.7%), and obese (34.9%). Income level distribution was as follows: 21.8% poverty, 19% low, 26.5% middle, and 32.7% high income (Table 1).

**Figure 2:**
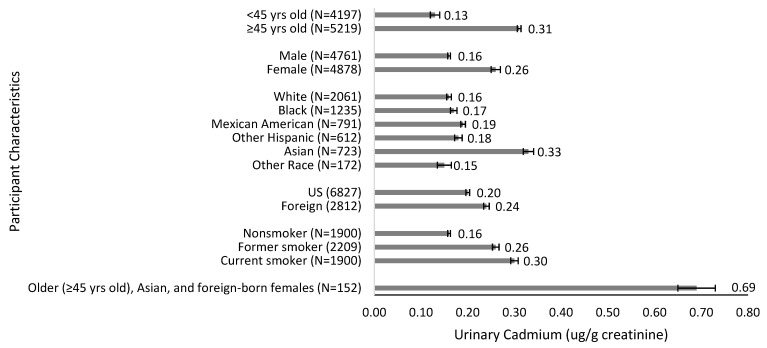
Geometric mean value of urinary cadmium levels (µg/g creatinine) by age group, gender, race/ethnicity, nativity, and smoking status.

Older participants (≥45) had significantly higher urinary cadmium levels compared to the younger group (0.31 µg/g vs. 0.13 µg/g; *p* < 0.001). Females had higher cadmium levels compared to males (0.26 µg/g vs. 0.16 µg/g; *p* < 0.001). Regarding race, Asians had the highest cadmium levels (0.33 µg/g), followed by Mexican Americans (0.19 µg/g), other Hispanics (0.18 µg/g), Blacks (0.17 µg/g), and Whites (0.16 µg/g) (*p* < 0.001). Cadmium levels were higher in foreign born participants at 0.24 µg/g compared to 0.20 µg/g in U.S. born subjects (*p* < 0.001). Compared to nonsmokers, former smokers and current smokers had increased urinary cadmium levels (0.16 µg/g vs. 0.26 µg/g and 0.30 µg/g; *p* < 0.001) (see Table 2, Figure 2).

We divided our study into five time periods based on NHANES 2-year cycles (2007–2008, 2009–2010, 2011–2012, 2013–2014, and 2015–2016). Cadmium levels decreased from 0.24 µg/g to 0.18 µg/g over the study period (*p* < 0.001). However, foreign (vs. U.S. born) individuals did not show a significant decrease over time. Moreover, cadmium levels decreased in all income groups (*p* < 0.001) except in the poverty group.

On multivariate analysis, older age (OR = 8.87, 95% CI: 7.48–10.53; *p* < 0.001), female sex (OR = 4.32, 95% CI: 3.59–5.21; *p* < 0.001), Asian race (OR = 4.52, 95% CI: 3.10–6.61; *p* < 0.001), and foreign-born status (OR = 1.83, 95% CI: 1.48–2.26; *p* < 0.001) were associated with higher urinary cadmium levels. In addition, poverty (OR = 1.35, 95% CI: 1.08–1.68; *p* = 0.01) and smoking (OR = 5.74, 95% CI: 4.69–7.02; *p* < 0.001) were independent predictors of cadmium exposure. Compared to underweight status, obesity was associated with lower cadmium levels (OR = 0.66, CI: 0.42–1.03; *p* = 0.002) (see Table 3).

In a separate analysis to calculate trend-test on time-period while adjusting for age, gender, nativity status, race/ethnicity, income, BMI, smoking status, and longest occupation, we treated time-period as a numeric value. On trends analysis, there was a decrease in cadmium levels over time (OR = 0.7847, 95% CI: 0.7339–0.8357; *p* < 0.001). On intersection analysis, we found that older Asian women had higher cadmium levels (0.50 µg/g) compared to young Asian women (0.32 µg/g). After including nativity status, our results showed that older, Asian, and foreign-born females had the highest urinary cadmium levels at 0.69 μg/g, approximately three-fold higher compared to the overall study group at 0.20 μg/g.

## 4. Discussion

It is well established that exposure to cadmium predisposes to serious illnesses across multiple organs including renal disease and cancers [15,16]. In this current report, our results suggest that older age, female sex, smoking, and poverty were associated with higher urinary cadmium levels. Furthermore, blue-collar work and the subgroup of older Asian foreign-born females had significantly greater risk. Over the 10-year study period, cadmium levels decreased across all groups except for those who had low socioeconomic status.

Similar to other reports, we found that older individuals had higher cadmium levels compared to younger people [7,8]. Since cadmium is an environmental pollutant that accumulates in the kidney over years, urinary cadmium can accurately measure long-term exposure, particularly in older individuals. We also found that females had higher cadmium levels compared to males [17]. This observed gender difference may be explained by the diminished iron stores, particularly in pre-menopausal women, leading to increased cadmium absorption [18,19]. Iron depletion leads to an upregulation of divalent metal transporter 1, increasing the absorption of iron and other metals including cadmium [20].

In reproductive-age women, investigators have found that Blacks have higher levels of cadmium compared to Whites [9]. However, others have shown that Asians and Hispanic Americans have the greatest cadmium exposure [21]. While these findings are valuable, they resulted from a smaller sample size than the current study, utilized blood cadmium as a measurement, and did not adjust for the range of factors accounted for in our current study. Our result showed that Asians are at greatest risk of elevated cadmium levels, which may be explained by dietary differences. Rice-heavy diets such as those found in several Asian cultures can act as significant cadmium sources [22,23]. While we were unable to identify dietary differences in these racial groups, it is possible that diet may be contributing to the disparities observed.

Previous studies have arrived at conflicting conclusions regarding the role of socioeconomic status on cadmium levels. Although our study showed a significant association between urinary cadmium levels and poverty, others did not find any relationship between cadmium and income level [17]. Jobs in steel industry plants and waste incineration have been shown to be major sources of cadmium exposure [24]. One study noted elevated levels in those working in mining, paper and wood, food, and the tobacco industries, and health care. It is likely that low-paying occupations with exposure to hazardous materials present the greatest risk of increasing cadmium burden [25].

Using an intersectional analysis, we found participants identified as older in age, female in sex, foreign-born, and Asian in race had the highest urinary cadmium levels. High levels of cadmium are associated with an increased risk in osteoporosis. Since older Asian women have a higher risk of low bone mineral density decline, these findings may have significant implications toward this at-risk subgroup [26]. Chronic, low-level cadmium exposure can not only lead to bone density reductions, but also early menopause and premature ovarian insufficiency [3,4].

This is one of the first large NHANES database studies to account for a wide range of demographic characteristics including all U.S. adults and incorporated a trends analysis. The current study also quantified cadmium burden using urinary measurements, a more accurate reflection of long-term cadmium exposure compared to blood levels due to renal accumulation. It also provides an intersectionality analysis that identifies older, foreign-born Asian subgroups at greater risk, a quality not found in existing studies [27]. Further studies will need to be performed to assess whether any strategies are effective in decreasing the cadmium exposure and associated adverse health outcomes.

Our study did not explore other sources of cadmium exposure. For example, we did not evaluate dietary information from each participant. It is well known that although rice is one of the highest sources of dietary cadmium, green leafy vegetables, potatoes, celery, carrots, wheat, and shellfish also contain high levels of cadmium [15]. This is likely due to the fact that cadmium in the soil is absorbed by plants. In addition, other sources of cadmium exposure include industrial paints, fertilizers, burning fossil fuels, and incineration of waste materials [2]. This study was also limited by our inability to assess duration of cadmium exposure (for example, how long a former smoker smoked). Furthermore, our study assessed cadmium associations with the “longest held occupation” but did not assess the duration of occupation.

## 5. Conclusions

Our study showed that Asians had the highest urinary cadmium levels, especially older, foreign-born females. Smoking and poverty were also associated with significant elevations in urinary cadmium levels. These results have potential implications toward influencing environmental policy to decrease exposure to environmental pollutants. With the advent of the “quantified self”, and new technology that allows for the self-tracking of environmental and other exposures, further studies are warranted to better understand the human exposome (chemical, biological, and physical exposures over a lifetime) in relation to cadmium and other pollutants in high-risk populations [28].

## Figures and Tables

**Table 1:** Longest occupation categories based on similar job types held by the participants. These definitions of categories and the examples were modified and grouped together from the NHANES database [11], as presented by Wei et al. [13] (the following table is presented with the authors’ permission).

Occupation Category	Occupation Types
White-collar	Management occupations, legal occupations
Science and education	Computer and mathematical occupations, architecture and engineering occupations, life physical, science and social science, education training and library occupations
Health-related	Health care practitioner technical occupations, health support, personal care service occupations
Sales, finance, business-related	Business, financial operations occupations, arts design entertainment sports media occupations, sales and related occupations
Office, administrative support	Community social services occupations, office and administrative support occupations
Food preparation and service	Food preparation service occupations
Blue collar	Protective services, building and cleaning maintenance occupations, farming fishing forestry occupations, construction extraction occupations, installation repair maintenance occupations, production occupations, transportation material moving occupations, armed forces

**Table 2:** Association between the patients’ characteristics and geometric mean values of urinary cadmium levels.

Characteristics	Overall (N = 9639)	Cadmium (µg/g Creatinine)		
	N(%)	Geometric Mean	Range	*p*-Value
Time-Period				<0.001 ^a^
2007–2008	1934 (19%)	0.24	0.01–4.04	
2009–2010	2111 (20%)	0.23	0.01–3.57	
2011–2012	1805 (20%)	0.21	0.02–7.56	
2013–2014	1925 (21%)	0.17	0.01–5.49	
2015–2016	1864 (21%)	0.18	0.01–2.73	
Age Group				<0.001 ^a^
Median (Range)	45 yrs (18–80)			
<45 yrs	4197 (47%)	0.13	0.01–7.56	
≥45 yrs	5219 (53%)	0.31	0.01–5.49	
Nativity				<0.001 ^a^
US	6827 (82%)	0.20	0.01–7.56	
Foreign	2812 (18%)	0.24	0.01–5.49	
Race/Ethnicity (2007–2016)				<0.001 ^a^
White	3933 (66%)	0.21	0.01–7.56	
Black	2029 (12%)	0.20	0.01–3.57	
Mexican American	1524 (9%)	0.17	0.01–5.49	
Other Hispanic	1059 (6%)	0.18	0.01–3.16	
Other ^b^	1094 (8%)	0.27	0.01–4.47	
Race/Ethnicity (2011–2016) ^c^				<0.001 ^a^
White	2061 (65%)	0.16	0.01–7.56	
Black	1235 (11%)	0.17	0.01–2.69	
Mexican American	791 (9%)	0.19	0.01–5.49	
Other Hispanic	612 (6%)	0.18	0.01–2.58	
Asian	723 (5%)	0.33	0.02–4.47	
Other ^d^	172 (3%)	0.15	0.01–0.91	
Gender				<0.001 ^a^
Male	4761 (48%)	0.16	0.01–5.49	
Female	4878 (52%)	0.26	0.01–7.56	
Income Level				<0.001 ^a^
Poverty	2873 (22%)	0.22	0.01–5.49	
Low	2342 (19%)	0.21	0.01–5.90	
Middle	2289 (27%)	0.21	0.01–4.47	
High	2135 (33%)	0.20	0.01–7.56	
Body Mass Index Category				<0.001 ^a^
Median (Range)	27 kg/m^2^ (13–83)			
Underweight	290 (3%)	0.30	0.02–3.84	
Normal	2781 (30%)	0.21	0.01–4.47	
Overweight	3104 (33%)	0.21	0.01–7.56	
Obese	3464 (35%)	0.20	0.01–4.04	
Smoking Status				<0.001 ^a^
Nonsmoker	5530 (56%)	0.16	0.01–7.56	
Former smoker	2209 (24%)	0.26	0.01–5.49	
Current smoker	1900 (20%)	0.30	0.01–5.90	
Longest Occupation ^e,f^				<0.001 ^a^
Never worked	392 (4%)	0.18	0.02–4.04	
White-collar	549 (9%)	0.20	0.01–5.90	
Science and education	658 (11%)	0.20	0.01–4.47	
Health-related	826 (11%)	0.24	0.01–4.22	
Sales, finance, business	1057 (15%)	0.20	0.01–7.56	
Office, administrative	980 (13%)	0.24	0.02–3.57	
Food preparation and services	548 (6%)	0.18	0.02–3.72	
Blue-collar	2736 (31%)	0.22	0.01–5.49	

^a^ Analysis of variance (ANOVA) was used to determine the difference in geometric mean of the log transformed creatinine-adjusted urinary cadmium levels between subgroups for each factor. ^b^ During 2007–2016, other race was reported as Asian race, mixed race, and other race not specified in the National Health and Nutrition Examination Survey (NHANES) database. ^c^ Asian race was collected by NHANES starting in 2011. ^d^ During 2011–2016, other race was reported as Asian race, mixed race, and other race not specified in the National Health and Nutrition Examination Survey (NHANES) database. ^e^ Data on detailed occupation was only available until 2014. ^f^ Longest occupation can be interpreted as either the current job or occupation that was held the longest.

**Table 3:** Multivariate logistic regression on the likelihood of high exposure risk of urinary cadmium levels, ≥2 µg/g, while adjusting for participant characteristics (2007–2014).

Characteristics	Odds Ratio	95% Confidence Interval	*p*-Value
Time-Period			
2007–2008	1		
2009–2010	0.88	0.71–1.09	0.23
2011–2012	0.72	0.57–0.90	0.005
2013–2014	0.47	0.39–0.57	<0.001
Age Group			
<45 yrs	1		
≥45 yrs	8.87	7.48–10.53	<0.001
Nativity			
US	1		
Foreign	1.83	1.48–2.26	<0.001
Race/Ethnicity ^a^			
White	1		
Black	1.17	0.91–1.52	0.21
Asian	4.52	3.10–6.61	<0.001
Mexican American	1	0.62–1.62	0.99
Other Hispanic	0.81	0.55–1.18	0.26
Other ^b^	1.12	0.52–2.44	0.76
Gender			
Male	1		
Female	4.32	3.59–5.21	<0.001
Income Level			
High	1		
Poverty	1.35	1.08–1.68	0.01
Low	1.17	0.95–1.44	0.14
Middle	1.18	0.97–1.44	0.09
Body Mass Index Category			
Underweight	1		
Normal	0.96	0.62–1.47	0.84
Overweight	0.81	0.53–1.25	0.33
Obese	0.66	0.42–1.03	0.07
Smoking Status			
Nonsmoker	1		
Former smoker	3.23	2.65–3.95	<0.001
Current smoker	5.74	4.69–7.02	<0.001
Longest Occupation ^c,d^			
Never worked	1		
White-collar	0.97	0.61–1.53	0.89
Science and education	0.88	0.59–1.32	0.54
Health-related	0.93	0.61–1.40	0.71
Sales, Finance, business	0.99	0.70–1.42	0.97
Office, administrative	0.997	0.71–1.39	0.99
Food preparation and services	0.74	0.52–1.06	0.10
Blue-collar	1.37	0.95–1.98	0.09

^a^ Asian participant data only available at the beginning in 2014, therefore Race/Ethnicity section reports the 2011–2014 data statistics. ^b^ Other race includes mixed race, and other race not specified in the National Health and Nutrition Examination Survey (NHANES) database. ^c^ Data on detailed occupation was only available until 2014. ^d^ Longest occupation can be interpreted as either the current job or occupation that was held the longest.

## Data Availability

Publicly available datasets were analyzed in this study. The data can be found at https://wwwn.cdc.gov/nchs/nhanes/Default.aspx (accessed on 7 August 2021).
